# Controlled positioning of nanoparticles on a micrometer scale

**DOI:** 10.3762/bjnano.3.86

**Published:** 2012-11-20

**Authors:** Fabian Enderle, Oliver Dubbers, Alfred Plettl, Paul Ziemann

**Affiliations:** 1 Institute of Solid State Physics, Ulm University, D-89069 Ulm, Germany

**Keywords:** electron beam lithography, nanoparticles, positioning, self-assembling, unconventional lithography

## Abstract

For many applications it is desirable to have nanoparticles positioned on top of a given substrate well separated from each other and arranged in arrays of a certain geometry. For this purpose, a method is introduced combining the bottom-up self-organization of precursor-loaded micelles providing Au nanoparticles (NPs), with top-down electron-beam lithography. As an example, 13 nm Au NPs are arranged in a square array with interparticle distances >1 µm on top of Si substrates. By using these NPs as masks for a subsequent reactive ion etching, the square pattern is transferred into Si as a corresponding array of nanopillars.

## Introduction

Nanoparticles (NPs) still play a major role in nanoscience from both an application and a fundamental point of view. Common to both aspects is the interest in possible new properties when reducing the sample size of a material down to the nanoscale. Quite generally, all material properties display in practice such size effects, while not all of them are advantageous for applications. An example for the latter case is provided by magnetic NPs, which for smaller and smaller particle volumes start exhibiting strong directional fluctuations in their magnetization and, thus, render their use for magnetic storage impossible at ambient temperature. On the other hand, this superparamagnetism poses experimental challenges to try and test new materials and alternative arrangements or novel concepts on the nanoscale to satisfy high-density magnetic data storage [1–4]. In this context, percolating magnetic media may be mentioned or “race track” arrangements, both relying on well-defined and positioned pinning sites for magnetic domain walls [5–6]. In a magnetic thin film, such pinning could be realized by local holes (“antidots”) leading immediately to quite a different application of NPs: using them as masks for subsequent etching procedures to transfer the NP pattern into their supporting substrate. In this respect, the notion of a nanoparticle should include as well colloids and micelles since their use for patterning is more widely spread [7–12]. Of course, in addition to their magnetic behavior, NPs offer attractive optical [13–14] or electrical [15–16] properties. In these cases, NPs fabricated from the complete spectrum of materials, i.e., insulators, semiconductors and metals are required. As a consequence, preparational progress in that field is still of utmost importance [17–18]. Assuming that a fabrication recipe has been developed for NPs of a desired material, there is, however, for many applications still another demanding requirement: positioning the NPs at predesigned locations, either with respect to geometry, such as forming squares or triangles, or, at least, with respect to interparticle distances, or even both. Restricting these distances to the nanoscale as well, some self-organization approaches exist that exploit hierarchical structure formation, allowing at least partial fulfillment of the above requirements [19–22]. For interparticle distances of some tens of nanometers creative ideas have been realized based on even three-dimensional DNA spacers linked to Au NPs [23]. Somewhat more flexible with respect to the type of NPs is their positioning, exploiting wettability contrast of a substrate previously prepared by, e.g., microcontact printing [24–25] or improved direct nanoscale embossing [26]. Though, in this case, the interparticle distances can be largely enhanced, the difficulty here is to avoid obtaining more than one particle at a given location. For interparticle distances of some hundred nanometers colloidal approaches have been successfully demonstrated. Though related to two-dimensional non-close-packed colloidal crystals [11] and, thus, primarily leading to the formation of hexagonal arrays of NPs, the method is novel in that it applies colloids carrying metal precursors. Once the colloidal carriers form a self-assembled ordered array, plasma processes are exploited to remove the organic matrix and to reduce the precursors into metallic NPs [10,12]. Though this technique appears quite versatile with respect to the type of NPs, it still has restrictions related to geometries other than hexagonal symmetry and distances well above 1 µm. It is exactly this problem of combining the nano- with the micro-scale that is the focus of the present contribution. In the following approach, NPs prepared by exploiting the self-organization of precursor loaded micelles formed from diblock-copolymers play a major role as a starting point. Thus, the genuine symmetry of their original arrangement again will be hexagonal. However, as will be demonstrated below, combining the micellar method with conventional electron-beam lithography not only extends interparticle distances from typically 100 nm into the micrometer range, but additionally allows a broad variation of geometries for the finally arranged NPs.

## Results and Discussion

### Preparation of Au nanoparticles (NPs)

**Figure 1:**
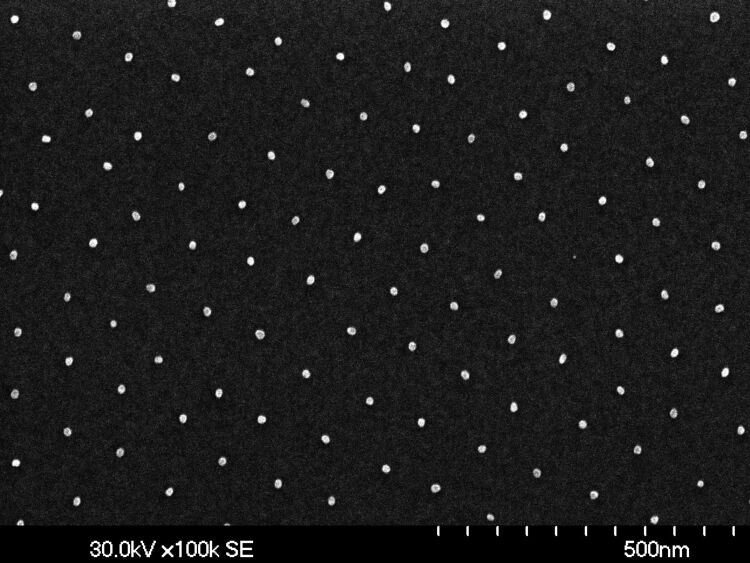
SEM image of Au nanoparticles (average diameter 13 nm, interparticle distance 102 nm) deposited on top of a Si substrate by applying an approach based on self-organization of precursor-loaded reverse micelles.

The starting point of the present approach is the fabrication of hexagonally arranged Au NPs applying a previously reported recipe based on the self-organization of precursor-loaded micelles [7–8,21]. In short, commercially available *diblock*-copolymers [polystyrene-block-poly-2-vinylpyridine (PS-b-P2VP) from Polymer Source Inc, Canada] forming spherical reverse micelles in an apolar solvent, such as toluene, are loaded with HAuCl_4_ salt as precursor. After optimized dip coating of the substrate (presently n-doped (001)-oriented Si wafers; in general, however, any reasonably flat substrate material is suitable), one single layer of hexagonally ordered micelles is obtained. By exposing such micellar layers to a hydrogen plasma the organic species can be completely removed and the precursor can be reduced to metallic Au NPs. The most attractive features of this approach are the control over the size of the NPs (determined by the amount of added precursor) as well as over the interparticle distance (determined by the total length of the *diblock*-copolymer and the substrate velocity during dip coating [8]). Furthermore, and most important for the present work, the final position of the Au NPs mirrors the self-assembled hexagonal array of the micellar carriers. This is demonstrated by the SEM image given in Figure 1 showing a typical array of Au NPs on top of a Si substrate.

The high degree of hexagonal order is clearly visible, although deviations from perfect order are obvious as well. In the present work, exclusively Au NPs with average diameters of 13 ± 1.6 nm were used. Smaller Au NPs, however, with diameters down to 2 nm would be easily available. Also, the interparticle distance was fixed at an average value of 102 ± 3 nm, for reasons to be discussed further below.

### Selecting Au nanoparticles on the micrometer scale

**Figure 2:**
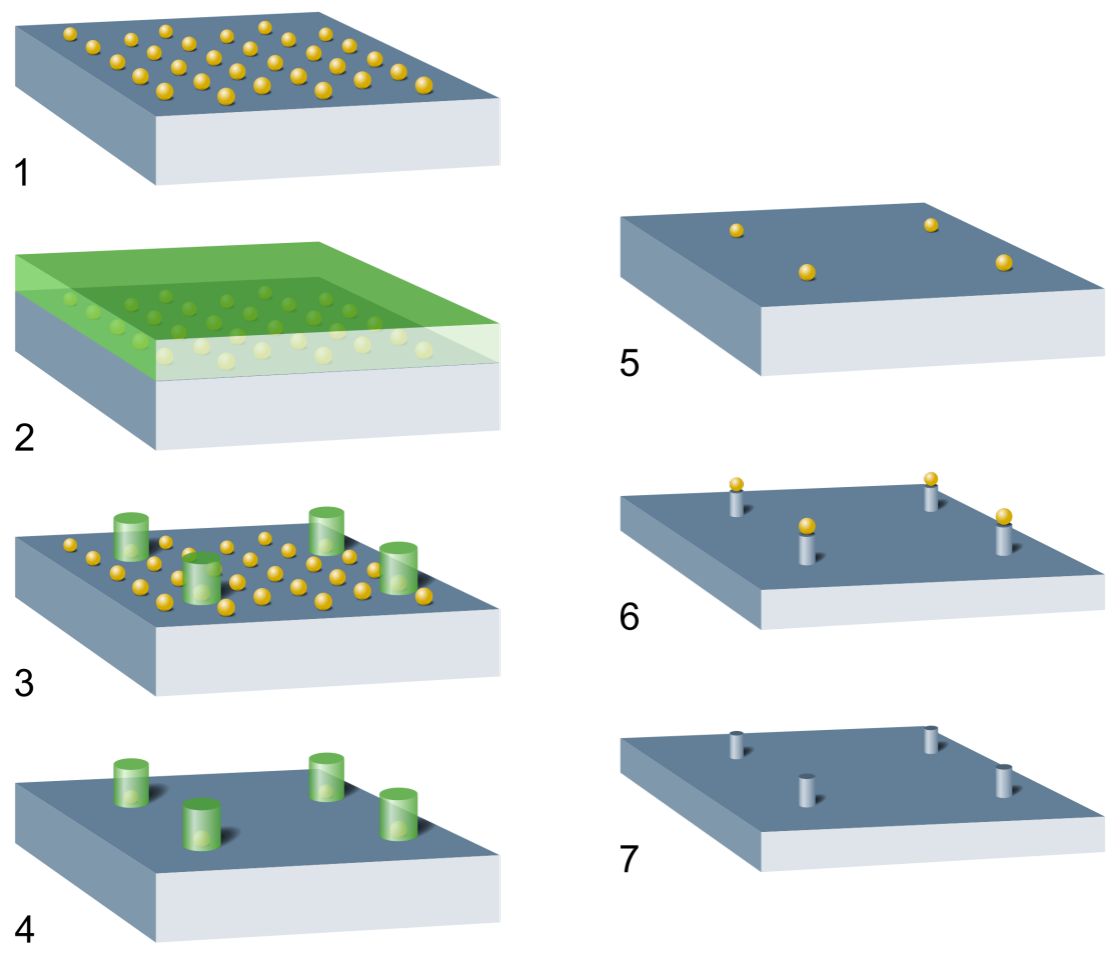
Schematics of the process leading to positioning nanoparticles on the micrometer scale: (1) Start; nanoparticles (NP) prepared by applying a method based on the self-organization of precursor loaded micelles on top of a flat substrate; here Au NP on Si; (2) Spin-coated negative resist for electron-beam lithography (EBL); (3) Resist disks arranged in a square lattice as obtained after EBL; (4) Removal of the residual Au NP between the disks by I/KI solution; (5) Stripping of the resist delivers the final NP arrangement; (6) Optional; using the NP as masks for a subsequent RIE etching step resulting in correspondingly arranged nanopillars; (7) Optional; removing the residual NP masks.

The basic idea behind selecting individual Au NPs on the micrometer scale is outlined by the schematics presented in Figure 2.

**Figure 3:**
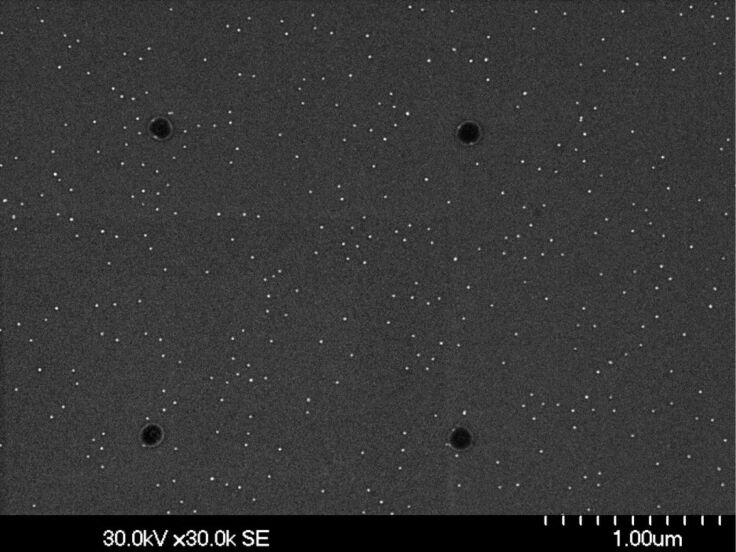
SEM image of resist disks arranged in a square (dark contrasts) as obtained after development (step 3 in Figure 2). The bright dots image the still present residual Au NPs, which have completely lost their hexagonal order during removal of the unexposed resist.

A negative resist (AR-N7500-18, Allresist, 6000 rpm, thickness approximately 300 nm) is spin coated above the primarily deposited Au NPs. Prior to this step, it is important to give the Si substrate with the NPs a short HF dip (2% HF, 10 s), which significantly enhances the adhesion of the resist. After a standard prebake of the resist (60 s at 85 °C on a hot plate), a square arrangement of circles is written into the resist by an electron beam (20 kV, 15 pA). The diameter of these circles has to be adjusted with respect to the interparticle distance of the Au NPs since each written resist disk should cover just one single NP. For the presently used mutual particle distance of 100 nm, a diameter of the resist disks of also 100 nm was chosen. This choice is the appropriate compromise to avoid having either no Au NPs covered by the circular resist island or more than one. By writing various square arrays of disks the optimum electron dose is determined, and the resist is thus developed (developer: 140–160 s, AR300-47 with water as stopper) followed by a postbake (80 s at 120 °C on a hot plate) of the exposed disks. The situation after this resist-removal step is illustrated by the SEM image shown in Figure 3. The four resist disks arranged in a square are clearly visible by their darker contrast while the bright dots image the residual Au NPs. Obviously, due to the development process the original hexagonal order of the NPs (Figure 1) is almost completely destroyed and some of the original Au NPs are even removed together with the unexposed resist.

**Figure 4:**
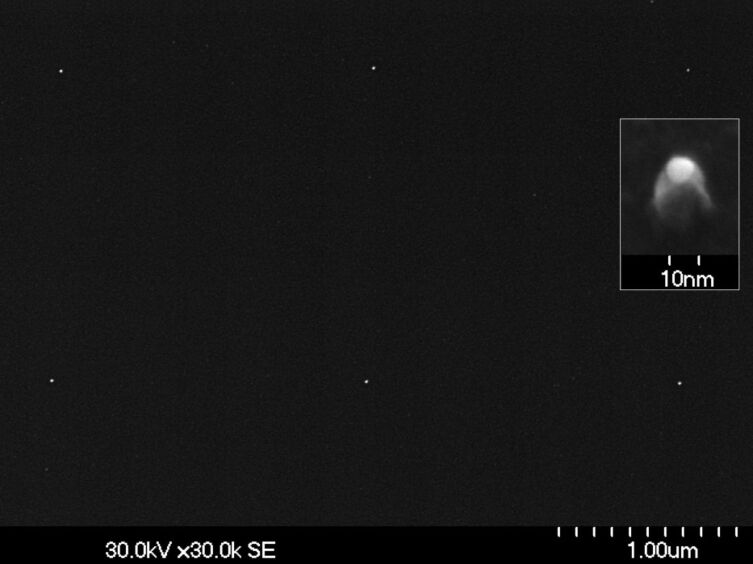
SEM image of two squares of nanopillars as obtained after RIE with single Au NPs, arranged in a square, as etching masks (step 5 in Figure 2). Distance between pillars: 1.8 μm. Inset: magnified SEM image (tilted by 30°) of one nanopillar with residual Au mask as cap.

Next, the residual uncovered Au NPs are removed by dipping the substrate into an I/KI solution for 30 s followed by the final stripping of the resist (1–2 min acetone, 20 s IPA). In principle, this last step finalizes the process delivering 13 nm Au NPs arranged in a square lattice with mutual distances in the micrometer range. However, to enhance visibility of these NPs in an overview SEM image, the particles are used as a mask during a subsequent reactive ion etching (RIE) of the Si substrate transforming the NPs into nanopillars. The result is demonstrated in Figure 4.

**Figure 5:**
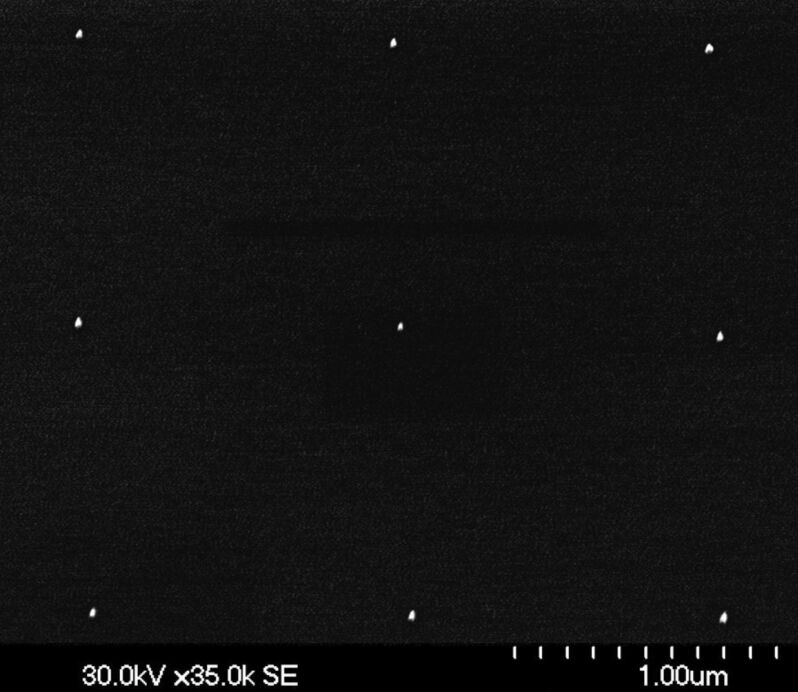
SEM image of four squares of nanopillars as obtained after RIE with single Au NPs, arranged in a square, as etching masks (step 5 in Figure 2). Distance between pillars: 1.2 μm.

Further squares of correspondingly prepared nanopillars can be visualized by reducing the interparticle distance from 1.7 μm in Figure 4 to 1.3 μm in Figure 5.

### Problems and compromises

Though the SEM images presented in Figure 4 and Figure 5 successfully deliver a proof of principle for the presently suggested positioning procedure, some problems should be addressed as well. The first point is related to the absolute precision of positioning the Au NPs. Writing any pattern such as the square array of disks by the electron beam is performed relative to a predetermined rectangular coordinate system fixed within the sample surface. When restricting the patterning to a 100 μm × 100 μm area, no mechanical movement of the sample holder is necessary, rather all programmed positions are approached by steering the electron beam. During the writing process, however, one observes a time-dependent drift, which in the present case of 100 nm disks arranged in squares added up to approximately 50 nm. Added to this error is the uncertainty of the exact position of the Au NP within any disk. Due to the finite hexagonal order, over larger areas this position can be assumed as random within the disk area. Thus, a very conservative estimate of the deviation of the NP location from an ideal square position is <150 nm, i.e., on the order of 10% in the present examples.

For many applications, however, positional precision of the NPs is not the primary goal. Rather, the NPs should be well separated from each other and individually identifiable against the background. Two classes of applications may illustrate these requirements. The first example is spectroscopy applied either directly to nanoparticles or, indirectly, on, e.g., molecules specifically ligated to the NPs, such as bonding to Au NPs through a thiol-group. To suppress interactions between nanoparticles or the molecules bound to them, usually interparticle distances of 50 nm are sufficient (for a recent study on near-field effects around a single dot see [27]). To guarantee single particle/molecule spectroscopy significantly larger distances are necessary as provided by the present method, depending in detail on the wavelength of the exciting radiation or the achievable focus size. In a second class of experiments, metallic NPs may be used as electrical contacts connected to the backside of the substrate by vias (vertical interconnect access), which, in turn, are further connected to pads on the micrometer scale. An example would be contacting a biological cell with typical lateral extensions of more than 10 µm at well-defined positions, e.g., 1 µm apart.

Though the presently obtained lateral precision of the particle positioning is sufficient for the just mentioned applications, further improvements appear possible. A necessary prerequisite for this would be a better long-range order of the starting NPs. For this, changing to self-assembled precursor-loaded colloids rather than micelles is promising [10–12]. In the ideal case, positioning of the resist disks would no longer be purely statistical but instead conform to multiples of the lattice parameter of the underlying hexagonal colloid lattice. To exploit the high long-range colloidal order, however, a sample holder with laser-interference-controlled translations becomes a must. In this way, positioning with a precision of better than 50 nm appears possible.

## Conclusion

A general procedure is introduced to position nanoparticles on the micrometer scale on top of a given substrate. The method is demonstrated for Au NPs (diameters 13 nm) on Si wafers in a square lattice with interparticle distances above 1 µm. The underlying idea is to combine the self-organization of precursor loaded micelles formed from *diblock*-copolymers in toluene, which is a bottom-up process providing nanoparticles, with top-down electron-beam lithography. As a first simple application, the resulting array of Au NPs is used as a mask for a subsequent reactive-etching process delivering correspondingly arranged Si nanopillars.
